# Draft Genome Sequence of a Polymyxin-Resistant Klebsiella pneumoniae Clinical Strain Carrying *mcr-8.1* and *bla*_NDM-5_

**DOI:** 10.1128/MRA.01224-20

**Published:** 2021-03-25

**Authors:** Na Pei, Zijuan Jian, Yirui Liu, Tianzhu Liang, Wenen Liu, Junhua Li

**Affiliations:** a BGI-Shenzhen, Shenzhen, China; b Department of Clinical Laboratory, Xiangya Hospital, Central South University, Changsha, Hunan, China; c Laboratory of Genomics and Molecular Biomedicine, Department of Biology, University of Copenhagen, Copenhagen, Denmark; d School of Basic Medicine, Qingdao University, Qingdao, Shandong, China; Indiana University, Bloomington

## Abstract

Carbapenem-resistant Klebsiella pneumoniae (CRKP) is a major threat to global health. Here, we report the draft genome sequence of a Klebsiella pneumoniae clinical strain carrying *mcr-8.1* and *bla*_NDM-5_.

## ANNOUNCEMENT

Infections caused by carbapenem-resistant Klebsiella pneumoniae (CRKP) have been increasing in recent years in hospitals and communities, which has attracted concern worldwide (1). Polymyxin is used as the last-resort antimicrobial drug for treating CRKP (2) infections. The *mcr* gene encodes the production of an enzyme which can alter the cell membrane of CRKP, enabling the bacteria to be resistant to polymyxin. However, a variety of *mcr* gene subtypes have been found since the first report of the plasmid-mediated colistin resistance gene *mcr-1* in 2015 (3–9). Studies show that the use of colistin has probably accelerated the dissemination of the *mcr* gene in animals and humans. K. pneumoniae strain 163109936 was recovered from the sputum sample of a 37-year-old male patient in the bone marrow transplantation department at a tertiary hospital in Hunan Province, China, in August 2016. The sputum was then plated and screened using sheep blood agar overnight. The MIC of colistin was determined using the broth microdilution method. The study was approved by the Human Ethics Committee of Xiangya Hospital of Central South University in Changsha, China (number 201806861). Strain 163109936 is resistant to ertapenem (MIC, ≥8 μg/ml), imipenem (MIC, ≥16 μg/ml), meropenem (MIC, 6 μg/ml), and polymyxin B (MIC, 8 μg/ml). The extensive drug resistance caught our attention. Therefore, this isolate was subjected to whole-genome sequencing.

The isolate was grown on LB broth at 37°C and harvested by centrifugation at 10,000 rpm for 1 min. Genomic DNA from strain 163109936 was extracted from the sample using a TIANamp bacterial DNA kit (Tiangen Biotech Co. Ltd., Beijing, China). The library was generated using the MGIEasy universal DNA library prep set (item number 1000006985). Sequencing was performed on a BGISEQ-500 sequencing platform (MGI, Shenzhen, China) with the paired-end 150-bp strategy. A total of 0.811 Gb was generated, and raw reads were filtered using fastp v 0.18.0 (10) and SOAPnuke (11) v 2.1.2 with default settings. The clean reads were assembled using SPAdes v 3.10.1 (12) with default settings. A total of 163 contigs (*N*_50_, 346,937 bp; *N*_90_, 34,594 bp; total length, 5,813,626 bp) were generated with 56.64% G+C content. The genome with a size of 5.8 Mb was annotated using Prokka v 1.14 (13) with default settings. Multilocus sequence typing (MLST) of K. pneumoniae was performed according to the protocol described on the Pasteur Institute MLST website (http://bigsdb.pasteur.fr/klebsiella/). The strain belongs to sequence type 685 (ST685), which has been found in China, Italy, and Turkey (14, 15).

The K. pneumoniae strain 163109936 genome contains 5,426 coding sequences and 88 tRNA genes. Antimicrobial resistance genes were identified using Resistance Gene Identifier v 4.0.3 (16) with default settings. According to our results, the strain was positive for several antimicrobial resistance genes, including *bla*_NDM-5_ (carbapenemase gene), *bla*_TEM-1_, and *bla*_CTX-M-65_. Most importantly, the colistin resistance gene *mcr-8.1* was also found. The presence of these genes could confer polymixin resistance, though this would need to be further validated. Plasmid replicons were also predicted with the presence of 10 replicons, IncFIB, IncFII, ColRNAI, IncR, Col, IncFIA, IncX3, TrfA, IncHI2A, and IncHI2.

In conclusion, a polymyxin-resistant Klebsiella pneumoniae clinical strain carrying *mcr-8.1* and *bla*_NDM-5_ was characterized. This reminds us that continuous monitoring of polymyxin resistance is urgent in clinical practice.

### Data availability.

This draft genome sequence has been deposited at GenBank under the accession number JACJVA000000000. The version described in this paper is version JACJVA010000000. The raw reads have been submitted to the Sequence Read Archive (SRA) database under the accession number SRR12995617.
